# Supplementary material for: HIVprotI: an integrated web based platform for prediction and design of HIV proteins inhibitors
Source: J Cheminform. 2018 Mar 9;10:12. doi: 10.1186/s13321-018-0266-y (PMC5845081; doi:10.1186/s13321-018-0266-y)
Supplement: Supplementary file 2 — Additional file 2. Source code of HIVProtI web server. [file 13321_2018_266_MOESM2_ESM.zip › HIVprotI_Source-code/hivprotI/jsmol/test2.htm]

HTML5-only and WebGL Jmol Demo


For a zip file containing all the files in this directory,
see http://chemapps.stolaf.edu/jmol/zip/Jmol.zip.
This test page uses unminified files and can implement the signed Jmol applet, the unsigned Jmol applet, or just JavaScript and HTML5 with or without WebGL.
Credits:
WebGL interface written by **Takanori Nakane**.
Java2Script written by **Zhou Renjian, et al.**
Jzlib written by **Atsuhiko Yamanaka**.
Jmol code conversion to JavaScript by **Bob Hanson**.

console
image
display
faster
sharper
  
platformSpeed:
8
7
6
5
4
3
2
1
  
info show
clear
hide
  
spacefill
wire
ball&stick
cartoons
fancy
not
flat
  
color atomno
color cpk
color structure
  
isosurface vdw
off
mep
translucent
opaque
  
labels on
off
echo
larger
smaller

**Try various buttons. If anything doesn't work please let me know.**
